# Metabolic Reprograming in Macrophage Polarization

**DOI:** 10.3389/fimmu.2014.00420

**Published:** 2014-09-02

**Authors:** Silvia Galván-Peña, Luke A. J. O’Neill

**Affiliations:** ^1^School of Biochemistry and Immunology, Trinity Biomedical Science Institute, Trinity College Dublin, Dublin, Ireland

**Keywords:** metabolism, macrophage, HIF, glycolysis, PGC-1β

## Abstract

Studying the metabolism of immune cells in recent years has emphasized the tight link existing between the metabolic state and the phenotype of these cells. Macrophages in particular are a good example of this phenomenon. Whether the macrophage obtains its energy through glycolysis or through oxidative metabolism can give rise to different phenotypes. Classically activated or M1 macrophages are key players of the first line of defense against bacterial infections and are known to obtain energy through glycolysis. Alternatively activated or M2 macrophages on the other hand are involved in tissue repair and wound healing and use oxidative metabolism to fuel their longer-term functions. Metabolic intermediates, however, are not just a source of energy but can be directly implicated in a particular macrophage phenotype. In M1 macrophages, the Krebs cycle intermediate succinate regulates HIF1α, which is responsible for driving the sustained production of the pro-inflammatory cytokine IL1β. In M2 macrophages, the sedoheptulose kinase carbohydrate kinase-like protein is critical for regulating the pentose phosphate pathway. The potential to target these events and impact on disease is an exciting prospect.

## Introduction

Early insights into the metabolic status of macrophages date back to the pioneering work carried out by G. C Hard more than 40 years ago. Hard showed that activated murine peritoneal macrophages had lower levels of oxygen consumption than resting ones as well as higher levels of glycolysis (1). This study provided the first evidence of a significant metabolic change in the macrophage as a consequence of activation. Studies by Newsholme and colleagues in the 1980s provided further evidence supporting this idea, as they were able to show that enzymes involved in glucose metabolism have higher enzymatic activities in macrophages, resulting in high rates of glucose and glutamine consumption (2).

Shortly afterward, in the early 1990s, a role for IL4 in macrophage activation was described, as well as the concept of alternative activation (3, 4). At this stage, a distinction was made between classically activated macrophages, also known as M1, and alternatively activated macrophages, also referred to as M2. M1 macrophages are activated by bacterial-derived products such as lipopolysaccharide (LPS), as well as by signals associated with infection such as IFNγ. This type of activation results in a highly inflammatory macrophage with high phagocytic and bactericidal potential. M2 macrophages on the other hand can be activated by parasitic products as well as signals associated with parasitic infections, such as the cytokines IL4 and IL13. This gives rise to a macrophage with anti-parasitic and tissue repair functions (5).

Also during this period, further research was carried out on the metabolic changes associated with macrophage activation. Bustos and Sobrino suggested for the first time that the inhibition of cytokine production in macrophages caused by glucocorticoids could be due to the inhibition of the glycolytic enzymes PFK1 and PFK2, thus directly implicating impaired metabolism with impaired function (6). A key discovery was, however, in arginine metabolism. Inés María Corraliza and colleagues were able to show that different enzymes responsible for the metabolism of arginine would be induced in a macrophage depending on the type of activation. In an M1 macrophage, nitric oxide synthase (iNOS) is upregulated, resulting in the catabolism of arginine to citrulline and nitric oxide, the latter playing key role in the intracellular killing of pathogens. In an M2 macrophage on the other hand, arginase-1 (Arg1) is induced, which results in the production of urea, polyamines, and ornithine, which are important for the wound healing actions of this macrophage population (7, 8). The differential metabolism of arginine is as of today, one of the most reliable discriminating factors between M1 and M2 macrophages. In fact, it is the only factor identified so far that can be used to detect M2 macrophage polarization in human samples (9). This provides an example of how studying the metabolic status of macrophages has proven more useful than studying function alone as well as unveiling the potential for therapeutic targeting in disease.

## Glycolytic M1 Versus Oxidative M2 Macrophages

Although studies into the metabolism of immune cells date back a few decades, it has only been in recent years that the tight link between metabolism and function has become apparent. The clear metabolic differences existing between M1 and M2 macrophages exemplify this idea. An M1 macrophage is part of the first line of defense of the innate immune system, which takes place within hours to days, as opposed to an M2 macrophage, which plays a bigger role within the resolution phase and thus has longer-term functions. Their metabolism is unsurprisingly a clear reflection of those functions.

**Figure 1:**
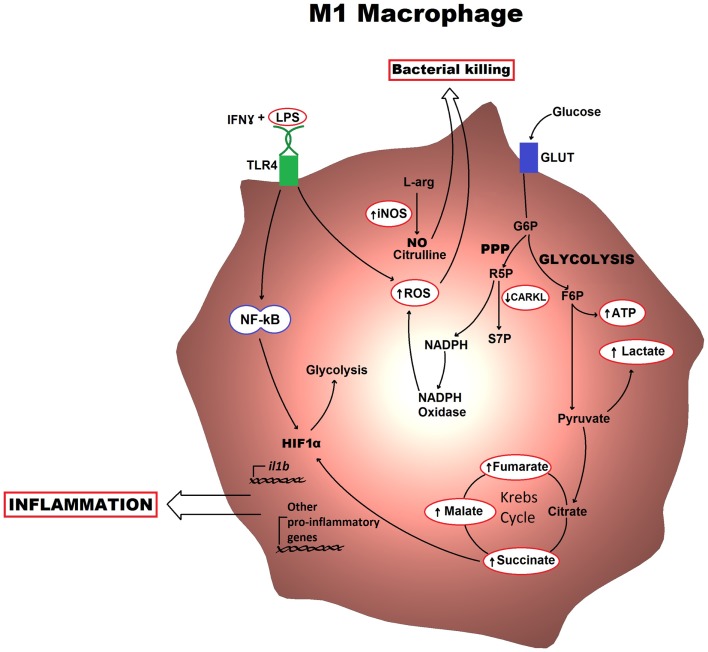
**Metabolic profile of an M1 macrophage is shown**. Classically activated macrophages induce an aerobic glycolytic program that results in lactate production and increased levels of intermediates of the Krebs cycle. The HIF1α transcription factor also becomes activated and can drive production of pro-inflammatory cytokines. The key functional consequences are bacterial killing, mostly through the production of ROS and NO, and inflammation, which occurs via cytokine production. G6P, glucose-6-phosphate; F6P, fructose-6-phosphate; R5P, ribulose-5-phosphate; S7P, sedoheptulose phosphate; NO, nitric oxide; ROS, reactive-oxygen species.

**Figure 2:**
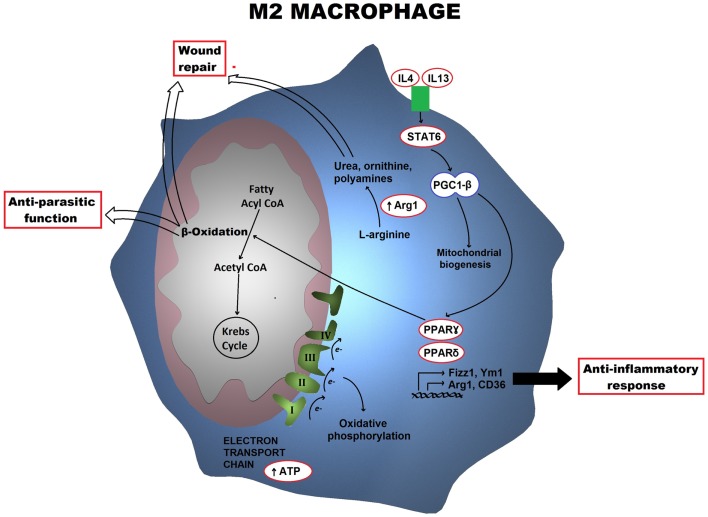
**Metabolic profile of an M2 macrophage is shown**. Alternatively activated macrophages trigger a metabolic program including the electron transport chain as well as fatty acid β-oxidation, which is orchestrated by STAT6 and PGC-1β. Arg1 also drives the production of polyamines and ornithine. The key functional consequences are tissue repair and anti-parasitic responses.

In M1 macrophages, aerobic glycolysis is induced upon activation, which involves an increase in glucose uptake as well as the conversion of pyruvate to lactate (Figure 1). At the same time, the activities of the respiratory chain are attenuated, allowing for reactive-oxygen species (ROS) production. Further evidence for this is provided when treating macrophages with the electron transport chain inhibitors rotenone and antimycin A as this mimics the effects of toll-like receptor (TLR) agonists in driving ROS production from the mitochondria (10). Furthermore, the pentose phosphate pathway is also induced following classical activation. This pathway is key for the generation of NADPH for the NADPH oxidase, which is important for ROS production, but also for nitric oxide synthesis (11). Altogether, these metabolic events can provide the cell with rapid energy and reducing equivalents, which are required for bactericidal activity. M2 macrophages on the other hand obtain much of their energy from fatty acid oxidation and oxidative metabolism, which can be sustained for longer. Following activation, they can induce expression of constituents of the electron transport chain that will perform oxidative phosphorylation as well as driving the pyruvate into the Krebs cycle (Figure 2). The pentose phosphate pathway is also more limited in M2 macrophages. Blocking oxidative metabolism not only blocks the M2 phenotype but also drives the macrophage into an M1 state. Similarly, forcing oxidative metabolism in an M1 macrophage potentiates the M2 phenotype (12, 13). These key metabolic differences between differentially activated macrophages are widely accepted; however, the switches responsible for orchestrating these different profiles at the molecular level remain largely unknown and how exactly the cell’s metabolic status regulates polarization is not yet well understood.

Following classical activation, there is a switch in the expression of 6-phosphofructose-2-kinase/fructose-2,6-bisphosphatase (PFK2) isoforms from the liver-form (L-PFK2) to the more active ubiquitous form (u-PFK2), leading to fructose-2,6-bisphosphaste accumulation, which pushes the glycolytic flux. This switching occurs at the transcriptional level with the L-PFK2 gene, *PFKB3*, being induced following activation (12). Additionally, there seems to be a requirement for downregulation of the carbohydrate kinase-like protein (CARKL) for the development of an M1 phenotype. CARKL catalyzes the production of sedoheptulose-7-phosphate, an intermediate of the pentose phosphate pathway (Figure 1). Besides expression levels of CARKL rapidly decreasing following classical activation, CARKL-expressing cells show defects in LPS-induced superoxide production. Furthermore, overexpression of CARKL results in a decrease in the production of pro-inflammatory cytokines in accordance with an M2 phenotype. Altogether, this would suggest that CARKL may help drive the macrophage metabolism toward increased pentose phosphate pathway activity and increased redox state, thus supporting M1 polarization (14). Finally, activation of macrophages with LPS results in increased levels of Krebs cycle intermediates such as succinate and malate. Succinate, in particular, was shown to drive IL1β production through HIF1α, a response that could be blocked by inhibition of glycolysis using 2-deoxyglucose (15). This exemplifies how the macrophage metabolism is not simply needed for providing the energy required but can also have a direct involvement in the transcriptional regulation of the immune response.

Following alternative activation, the *PFKB1* gene instead of the *PFKB3* is expressed, resulting in higher levels of the liver isoform of PFK2 and lower levels of fructose-2,6-bisphosphate. The lower glycolytic levels are compensated with an increase in oxidative phosphorylation. Following macrophage activation with IL4, there is massive induction of an oxidative metabolic program, ranging from fatty acid uptake and oxidation, to oxidative phosphorylation and mitochondrial respiration. The mechanism behind this increase is somewhat better understood than that of glycolysis in M1 macrophages. Following IL4 treatment, the transcription factor STAT6, which is responsible for mediating the transcriptional responses of this cytokine, becomes activated. Active STAT6 can induce the coactivator protein peroxisome proliferator-activated receptor (PPAR)γ-coactivator-1β (PGC-1β). PGC-1β can induce mitochondrial respiration as well as mitochondrial biogenesis. Furthermore, together with the transcription factors, nuclear respiratory factor 1 (NRF-1) and estrogen-related receptor-α (ERRα), it drives the production of key mitochondrial components, such as cytochrome *c* and ATP synthase (16, 17). It is therefore not surprising that PGC-1β is considered as the key player responsible for the metabolic switch in M2 macrophages (Figure 2). In fact, knockdown of PGC-1β impairs not only the metabolic profile of M2 macrophages but also their functions (13). Furthermore, while PGC-1β is the key trigger, PPARs, particularly PPARγ and PPARδ, have a key role in maintaining the phenotype. PPARδ is responsible for orchestrating the effector functions of alternative activation, for instance, expression of the macrophage galactose-type C-type lectin 1 (MGL-1) as well as costimulatory molecules and other factors involve in the anti-inflammatory response. PPARγ on the other hand, is involved in the transcription of different factors required for β-oxidation of fatty acids (18, 19).

Recently, the protein TNF-alpha-induced protein 8-like 2 (TIPE2) has also been associated with an M2 phenotype, through the induction of arginine metabolism, which as already mentioned, is the most distinguished metabolic feature of M2 macrophages. Interestingly, TIPE2 exerts such function following long-term classical activation of macrophages with LPS and not alternative activation. Thus, TIPE2 uses the switching to arginine metabolism to negatively regulate inflammation, and can therefore re-program a classically activated macrophage into its anti-inflammatory counterpart (20).

## Hypoxia-Inducible Factor in Macrophage Polarization

Macrophages, as well as other immune cells, are usually found in inflamed sites, which are characterized by low oxygen levels. The transcription factor HIF thus plays an important role as one of the key mediators in the adaptation of macrophages to hypoxic conditions. This heterodimeric protein is composed of two subunits, an α and a β subunit. Three isoforms of the oxygen-sensitive α subunit have been identified. The HIF1α isoform is expressed ubiquitously, and is tightly linked to the inflammatory response and microbicidal activities. HIF2α on the other hand, is expressed in a more limited fashion, but it is present in myeloid cells (21, 22). There is evidence in the literature suggesting a role for the two HIFα isoforms, 1 and 2, in macrophage polarization. While HIF1α has been associated with classical macrophage activation, HIF2α has been recently linked to an M2 phenotype. These differential roles are, however, far from clear.

HIF1α expression can be driven by different classical activators through NF-κB, resulting in the production of pro-inflammatory cytokines and other mediators of the M1 phenotype, such as glycolytic enzymes and glucose transporters. HIF2α expression, on the other hand, occurs independently of NF-κB, which would be in accordance with alternative activation. Interestingly though, both isoforms seem to be important in maintaining levels of the NF-κB subunit p65 (23). A key mediator regulated by HIF1α is the M1 marker iNOS. Under hypoxic conditions, nitric oxide production through iNOS is HIF1α-dependent thus implicating HIF1α in bacterial clearance (24). In fact, HIF1α^−/−^ macrophages have impaired capacity to clear both Gram-positive and Gram-negative bacteria. Nevertheless, superoxide production during the respiratory burst, which is also required for bacterial clearance, seems to be a HIF1α-independent event (25). This is, interestingly, not the only HIF1α-independent event that occurs following classical activation. A critical event in the reprograming of metabolism to glycolysis is the switch from L-PFK2 to u-PFK2, which also occurs independently of HIF1α (12). This would suggest the presence of some other yet unidentified factor responsible for mediating the metabolic switch in M1 macrophages, either independently or in association with HIF1α.

The potential role of HIF2α in promoting the M2 phenotype, although promising, remains obscure. HIF2α has been shown to regulate transcription of the M2 marker, Arg1. This finding is supported by the half-life of both proteins, as both the mRNAs for HIF2α as well as Arg1, have relatively long half-lives. The mRNAs for HIF1α and iNOS, however, are relatively short-lived (24). This would agree with the initial idea of how the metabolism of polarized macrophages goes hand in hand with the timing of their functions, and would support the association of HIF1α and HIF2α with M1 and M2 macrophages, respectively. There are, however, incongruences regarding the role of HIF2α. For instance, HIF2α also controls IL1β production, which is associated with an M1 phenotype rather than M2 (15). Additionally, HIF2α has also been associated with NF-κB activity as mentioned above, which is also associated with an M1 phenotype (23). Studies have shown, however, that both isoforms seem to have redundant and overlapping functions, even though when one is knocked down, the other does not seem to be able to compensate (23). This highlights the fact that there are still major gaps in our understanding of the differential activities of the two isoforms.

## Polarization of Human Macrophages

Most current knowledge of macrophage polarization comes from murine studies; however, our understanding of this topic in human macrophages remains quite poor. Furthermore, the limited studies that have been carried out using human macrophages have identified major interspecies differences. For instance, classic murine M2 macrophage markers, such as Ym1 or Fizz1, lack human homologs and can therefore not be used as markers in human macrophages (26).

Interestingly, a recent proteomic analysis of differentially activated human macrophages suggests that the major functional differences between the two lie within metabolic pathways. The study identifies major metabolic enzymes such as glucose-6-phosphate dehydrogenase, fructose-1,6-bisphosphatase 1 (Fbp1), alpha enolase, and fructose bisphosphate aldolase A as being differentially expressed in human M1 and M2 macrophages (27). In agreement with murine studies, human M1 macrophages also upregulate glycolysis to give rise to a pro-inflammatory phenotype characterized by the production of cytokines such as IL12p40, TNFα, or IL6. However, oxidative metabolism and fatty acid oxidation do not seem to predominate in human M2 macrophages, but instead, gluconeogenesis, driven by Fbp1, seems to play a major role (27). This finding is supported by a subsequent study suggesting that fatty acid oxidation is dispensable in human M2 macrophages. D. Namgaladze and B. Brüne show that IL4-induced human M2 macrophages do not induce PGC-1β, the key transcription factor responsible for driving the fatty acid oxidation program. In contrast with murine studies, the use of a fatty acid oxidation inhibitor does not impair the ability of human macrophages to produce high levels of CCL18 and Mrc1 and low levels of IL1-β and IL6, suggesting that they maintain the M2 phenotype (28).

Another major aspect that seems to differ considerably between murine and human macrophages is the role of iNOS and Arg1 in M1 and M2 macrophages, respectively. Attempts to demonstrate significant production of NO by human macrophages in culture have mostly failed. When successfully detected, it has only been after a period of stimulation of a few days and in much smaller amounts than that detected in murine macrophages (29). Furthermore, a recent report indicates that epigenetic modifications silence the *nos2* gene in humans, suggesting that there is no role for iNOS in M1-mediated inflammation (30). Intriguingly, macrophage-derived NO production has been reported in cases of acute inflammation such as those presented by rheumatoid arthritis patients as well as those suffering from malaria (31, 32). On the other hand, it is not just the role of iNOS that has been questioned, but also that of Arg1. Neither Cameron et al. nor Sheemann et al. could detect any arginase activity from human macrophages in culture (33, 34). However, Anika Geelhaar-Karsch and colleagues have recently shown that patients suffering from classical Whipple’s disease, which is associated with elevated levels of M2 macrophages, present with higher levels of arginase activity as well as Arg1-derived products, such as urea (9). Interestingly, this could only be detected in plasma and fresh biopsies and not in macrophages in culture. Therefore, although the major differences existing between mice and humans in this regard are undisputable, the switch toward iNOS versus Arg1 may still play an important role in human diseases.

## Final Perspectives

The metabolic aspects behind macrophage activation have long been an area of interest for many. Metabolism as a key aspect of macrophage polarization, however, is an intriguing area within macrophage biology that has only started to develop more recently. Although we are still very much in the dark regarding our understanding of the metabolic molecular events driving macrophage polarization, the evidence discussed suggests that the role of metabolic intermediates is much more important than expected. The key question is why M1 and M2 macrophages would have such different metabolic profiles? It is possible that M1 macrophages are mainly found in hypoxic environments and therefore have to rely on glycolysis, produced via HIF1α, for their ATP production. Glycolysis can also be rapidly induced, which is perhaps needed for the rapid activation that occurs in M1 macrophages during infection. The attenuation in the respiratory chain will also allow M1 macrophages to produce ROS, as will the NADPH produced by the pentose phosphate pathway, which is required for the NADPH oxidase. For M2 macrophages, acute activation is less of an issue, as their main function is in wound healing and anti-parasitic defense. M2 macrophages also do not generate ROS and therefore have a fully functional respiratory redox chain, allowing for oxidation of fatty acids. β-oxidation of fatty acids has, in fact, been shown to be anti-inflammatory, possibly because of a decrease in the production of prostaglandins, although this is not fully understood (35). Perhaps, the more sustained role of M2 macrophages mainly involves the metabolism of fat reserves with less ROS being a safe-ground against injury during tissue repair.

The translation of these discoveries to human diseases is an intriguing prospect, especially, since there are diseases that have been associated with one particular macrophage phenotype or another. For instance, patients presenting with chronic venous ulcers suffer from chronic inflammation as a result of failing to switch from M1 macrophages to M2 (36). On the other hand, those suffering from classical Whipple’s disease, a result of chronic infection caused by *Tropheryma whipplei*, fail to clear the infection due to the lack of inflammation and excess presence of M2 macrophages (37). Interestingly, there are also reports suggesting that the distribution of M1 and M2 macrophages varies between males and females. In fact, the higher incidence of asthma in female mice was associated with higher levels of M2 macrophages when compared to male mice (38). Since females are known to present with higher incidence of not only asthma but also other autoimmune diseases, it would be interesting to speculate whether gender-associated differences in macrophage polarization might play a role.

Finally, manipulation of macrophage polarization has already proven to be somewhat successful clinically. Administration of the classical M1 macrophage activator IFNγ had beneficial effects in patients with ovarian carcinoma (39, 40). Therefore, our current understanding of the metabolic status of differentially activated macrophages holds great potential for clinical applications, although further research is required in order to capitalize clinically on the observations made to date in both murine and human systems.

## Conflict of Interest Statement

The authors declare that the research was conducted in the absence of any commercial or financial relationships that could be construed as a potential conflict of interest.

## References

[1] Hard GC Some biochemical aspects of the immune macrophage. Br J Exp Pathol (1970) 51(1):97–105 5434449 PMC2072214

[2] Newsholme P Curi R Gordon S Newsholme EA Metabolism of glucose, glutamine, long-chain fatty acids and ketone bodies by murine macrophages. Biochem J (1986) 239(1):121–5 3800971 10.1042/bj2390121 PMC1147248

[3] Abramson SL Gallin JI IL4 inhibits superoxide production by human mononuclear phagocytes. J Immunol (1990) 144(2):625–30 2153171

[4] Stein M Keshaw S Harris N Gordon S Interleukin 4 potently enhances murine macrophages mannose receptor activity: a marker of alternative immunologic macrophage activation. J Exp Med (1992) 176(1):287–92 10.1084/jem.176.1.287 1613462 PMC2119288

[5] Odegaard JI Chawla A Alternative macrophage activation and metabolism. Annu Rev Pathol (2011) 6:275–97 10.1146/annurev-pathol-011110-130138 21034223 PMC3381938

[6] Bustos R Sobrino F Stimulation of glycolysis as an activation signal in rat peritoneal macrophages: effect of glucocorticoids on this process. Biochem J (1992) 282:299–303 1311557 10.1042/bj2820299 PMC1130922

[7] Corraliza IM Soler G Eichmann K Modolell M Arginase induction by suppressors of nitric oxide synthesis (IL4, IL10, PGE_2_) in murine bone-marrow-derived macrophages. Biochem Biophys Res Commun (1995) 206(2):667–73 10.1006/bbrc.1995.1094 7530004

[8] Munder M Eichmann K Modolell M Alternative metabolic states in murine macrophages reflected by the nitric oxide synthase/arginase balance: competitive regulation by CD4^+^ T cells correlates with Th1/Th2 phenotype. J Immunol (1998) 160(11):5347–54 9605134

[9] Geelhard-Karsch A Schinnerling K Conrad K Friebel J Allers K Schneider T Evaluation of arginine metabolism for the analysis of m1/m2 macrophage activation in human clinical specimens. Inflamm Res (2013) 62:865–9 10.1007/s00011-013-0642-z 23775039

[10] West AP Brodsky IE Rahner C Woo DK Erdjument-Bromage H Tempst P TLR signaling augments macrophage bactericidal activity through mitochondrial ROS. Nature (2011) 472(7344):476–80 10.1038/nature09973 21525932 PMC3460538

[11] Aktan F iNOS-mediated nitric oxide production and its regulation. Life Sci (2004) 75(6):639–53 10.1016/j.lfs.2003.10.042 15172174

[12] Rodríguez-Prados JC Través PG Cuenca J Rico D Aragonés J Martín-Sanz P Substrate fate in activated macrophages: a comparison between innate, classic and alternative activation. J Immunol (2010) 185(1):605–14 10.4049/jimmunol.0901698 20498354

[13] Vats D Mukundan L Odegaard JI Zhang L Smith KL Morel CR Oxidative metabolism and PGC-1β attenuate macrophage-mediated inflammation. Cell Metab (2006) 4(1):13–4 10.1016/j.cmet.2006.08.006 16814729 PMC1904486

[14] Haschemi A Kosma P Gille L Evans CR Burant CF Starkl P The sedoheptulose kinase CARKL directs macrophage polarization through control of glucose metabolism. Cell Metab (2012) 15(6):813–26 10.1016/j.cmet.2012.04.023 22682222 PMC3370649

[15] Tannahill GM Curtis AM Adamik J Palsson-McDermott EM McGettrick AF Goel G Succinate is an inflammatory signal that induces IL-1β through HIF-1α. Nature (2013) 496(7444):238–42 10.1038/nature11986 23535595 PMC4031686

[16] St-Pierre J Lin J Krauss S Tarr PT Yang R Newgard CB Bioenergetic analysis of peroxisome proliferator-activated receptor gamma coactivators 1alpha and 1beta (PGC-1α and PGC-1β) in muscle cells. J Biol Chem (2003) 278(29):26597–603 10.1074/jbc.M301850200 12734177

[17] Shao D Liu Y Liu X Zhu L Cui Y Cui A PGC-1 beta-regulated mitochondrial biogenesis and function in myotubes is mediated by NRF-1 and ERR alpha. Mitochondrion (2010) 10(5):516–27 10.1016/j.mito.2010.05.012 20561910

[18] Odegaard JI Ricardo-Gonzalez RR Goforth MH Morel CR Subramanian V Mukundan L Macrophage-specific PPARγ controls alternative activation and improves insulin resistance. Nature (2007) 447(7148):1116–20 10.1038/nature05894 17515919 PMC2587297

[19] Kang K Reilly SM Karabacak V Gangl MR Fitzgerald K Hatano B Adipocyte-derived Th2 cytokines and myeloid PPARδ regulate macrophage polarization and insulin sensitivity. Cell Metab (2008) 7(6):485–95 10.1016/j.cmet.2008.04.002 18522830 PMC2586840

[20] Lou Y Zhang G Geng M Zhang W Cui J Liu S TIPE2 negatively regulates inflammation by switching arginine metabolism from nitric oxide synthase to arginase. PLoS One (2014) 9(5):e96508 10.1371/journal.pone.0096508 24806446 PMC4013027

[21] Cramer T Yamaniski R Clausen BE Förster I Pawlinski R Mackman N HIF1α is essential for myeloid cell-mediated inflammation. Cell (2003) 112(5):645–57 10.1016/S0092-8674(03)00154-5 12628185 PMC4480774

[22] Talks KL Turley H Gatter KC Maxwell PH Pugh CW Ratcliffe PJ The expression and distribution of the hypoxia-inducible factors HIF1α and HIF2α in normal human tissues, cancers, and tumor-associated macrophages. Am J Pathol (2000) 157(2):411–21 10.1016/S0002-9440(10)64554-3 10934146 PMC1850121

[23] Fang HY Hughes R Murdoch C Coffelt SB Bismas SK Harris AL Hypoxia-inducible factors 1 and 2 are important transcriptional effectors in primary macrophages experiencing hypoxia. Blood (2009) 114(4):844–59 10.1182/blood-2008-12-195941 19454749 PMC2882173

[24] Takeda N O’Dea EL Doedens A Kim JW Weidemann A Stockmann C Differential activation and antagonistic function of HIF-α isoforms in macrophages are essential for NO homeostasis. Genes Dev (2010) 24(5):491–501 10.1101/gad.1881410 20194441 PMC2827844

[25] Peyssonnaux C Datta V Cramer T Doedens A Theodorakis EA Gallo RL HIF1α expression regulates the bactericidal capacity of phagocytes. J Clin Invest (2005) 115(7):1806–15 10.1172/JCI23865 16007254 PMC1159132

[26] Raes G Van den Bergh R De Baetselier P Ghassabeh GH Scotton C Locati M Arginase-1 and Ym1 are markers for murine, but not human, alternatively activated myeloid cells. J Immunol (2005) 174(11):6561 author reply 6561-6562, 10.4049/jimmunol.174.11.6561 15905489

[27] Reales-Calderon JA Aguilera-Montilla N Corbi AL Molero G Gil C Proteomic characterization of human proinflammatory M1 and anti-inflammatory M2 macrophages and their response to *Candida albicans*. Proteomics (2014) 14(12):1503–18 10.1002/pmic.201300508 24687989

[28] Namgaladze D Brüne B Fatty acid oxidation is dispensable for human macrophage IL4-induced polarization. Biochem Biophys Acta (2014) 1841(9):1329–35 10.1016/j.bbalip.2014.06.007 24960101

[29] Albina JE On the expression of nitric oxide synthase by human macrophages. Why no NO? J Leukoc Biol (1995) 58(6):643–9 7499961 10.1002/jlb.58.6.643

[30] Gross TJ Kremens K Powers LS Brink B Knutson T Domann FE Epigenetic silencing of the human NOS2 gene: rethinking the role of nitric oxide in human macrophage inflammatory responses. J Immunol (2014) 192(5):2326–38 10.4049/jimmunol.1301758 24477906 PMC3943971

[31] St Clair EW Wilkinson WE Lang T Sanders L Misukonis MA Gilkeson GS Increased expression of blood mononuclear cell nitric oxide synthase type 2 in rheumatoid arthritis patients. J Exp Med (1996) 184(3):1173–8 10.1084/jem.184.3.1173 9064335 PMC2192765

[32] Anstey NM Weinberg JB Hassanali MY Mwaikambo ED Manyenga D Misukonis MA Nitric oxide in Tanzanian children with malaria: inverse relationship between malaria severity and nitric oxide production/nitric oxide synthase type 2 expression. J Exp Med (1996) 184(2):557–67 10.1084/jem.184.2.557 8760809 PMC2192721

[33] Cameron ML Granger DL Weinberg JB Kozumbo WJ Koren HS Human alveolar and peritoneal macrophages mediate fungistasis independently of L-arginine oxidation to nitrite or nitrate. Am Rev Respir Dis (1990) 142:1313–9 10.1164/ajrccm/142.6_Pt_1.1313 2123614

[34] Schneeman M Schoedon G Hofer S Blau N Guerrero L Shaffner A Nitric oxide synthase is not a constituent of the antimicrobial armature of human mononuclear phagocytes. J Infect Dis (1993) 167:1358–63 10.1093/infdis/167.6.1358 7684756

[35] Steinberg GR Schertzer JD AMPK promotes macrophage fatty acid oxidative metabolism to mitigate inflammation: implications for diabetes and cardiovascular disease. Immunol Cell Biol (2014) 92(4):340–5 10.1038/icb.2014.11 24638063

[36] Sindrilaru A Peters T Wieschalka S Baican C Baican A Peter H An unrestrained proinflammatory M1 macrophage population induced by iron impairs wound healing in humans and mice. J Clin Invest (2011) 121(3):985–97 10.1172/JCI44490 21317534 PMC3049372

[37] Moos V Schmidt C Geelhar A Kunkel D Allers K Schinnerling K Impaired immune functions of monocytes and macrophages in Whipple’s disease. Gastroenterology (2010) 138(1):210–20 10.1053/j.gastro.2009.07.066 19664628

[38] Melgert BN Oriss TB Qi Z Dixon-McCarthy B Geerlings M Hylkema MN Macrophages: regulators of sex differences in asthma? Am J Respir Cell Mol Biol (2010) 42(5):595–603 10.1165/rcmb.2009-0016OC 19574533 PMC2874445

[39] Allavena P Peccatori F Maggioni D Erroi A Sironi M Colombo N Intraperitoneal recombinant gamma interferon in patients with recurrent ascetic ovarian carcinoma: modulation of cytotoxicity and cytokine production in tumor-associated effectors and of major histocompatibility antigen expression on tumor cells. Cancer Res (1990) 50(22):7318–23 2121337

[40] Colombo N Peccatori F Paganin C Bini S Brandely M Mangioni C Anti-tumor and immunomodulatory activity of intraperitoneal IFN-gamma in ovarian carcinoma patients with minimal residual tumor after chemotherapy. Int J Cancer (1992) 51(1):42–6 10.1002/ijc.2910510109 1563843

